# Case report: Transition from anti-CD20 therapy to inebilizumab for 14 cases of neuromyelitis optica spectrum disorder

**DOI:** 10.3389/fneur.2024.1352779

**Published:** 2024-04-16

**Authors:** Benjamin Osborne, Gabriela Romanow, J. Michael Hemphill, Myassar Zarif, Tracy DeAngelis, Tyler Kaplan, Unsong Oh, Johnathan Pinkhasov, Kristina Patterson, Michael Levy

**Affiliations:** ^1^Department of Neurology, Georgetown University Medical Center, Washington, DC, United States; ^2^Department of Neurology, Massachusetts General Hospital and Harvard Medical School, Boston, MA, United States; ^3^Savannah Neurology Specialists, Savannah, GA, United States; ^4^South Shore Neurologic Associates, Patchogue, NY, United States; ^5^Neurological Associates of Long Island, New Hyde Park, NY, United States; ^6^Department of Neurology, Rush University Medical Center, Chicago, IL, United States; ^7^Department of Neurology, Virginia Commonwealth University, Richmond, VA, United States; ^8^Medical Affairs, Horizon Therapeutics, Deerfield, IL, United States

**Keywords:** neuromyelitis optica spectrum disorder, aquaporin-4 antibody, inebilizumab, rituximab, treatment transition, clinical outcome, case report

## Abstract

Neuromyelitis optica spectrum disorder (NMOSD) is a rare autoimmune disorder of the central nervous system characterized by recurrent, disabling attacks that affect the optic nerve, spinal cord, and brain/brainstem. While rituximab, targeting CD20-positive B-cells, is used as an off-label therapy for NMOSD, some patients continue to exhibit breakthrough attacks and/or adverse reactions. Inebilizumab, a humanized and glycoengineered monoclonal antibody targeting CD19-positive B-cells, has been FDA approved for the treatment of NMOSD in adult patients who are anti-aquaporin-4 (AQP4) antibody positive. Given the limited real-world data on the efficacy and safety of inebilizumab, especially in those transitioning from rituximab, a retrospective chart review was conducted on 14 NMOSD patients from seven centers. Of these, 71.4% (*n* = 10) experienced a combined 17 attacks during rituximab treatment, attributed to either breakthrough disease (*n* = 10) or treatment delay (*n* = 7). The mean duration of rituximab treatment was 38.4 months (3.2 years). Notably, no subsequent attacks were observed during inebilizumab treatment [mean duration of inebilizumab treatment was 19.3 months (1.6 years)], underscoring its potential as an effective treatment for NMOSD. Our data suggest that inebilizumab provides clinical benefit with effective disease control and a favorable safety profile for patients transitioning from rituximab.

## 1 Introduction

Neuromyelitis optica spectrum disorder (NMOSD) is a rare, debilitating autoimmune disorder of the central nervous system characterized by recurrent attacks affecting the optic nerve and spinal cord (1). However, NMOSD can also involve various other regions of the CNS, including the brainstem, area postrema, diencephalon, and cerebral white matter. NMOSD predominantly affects women and frequently leads to severe neurological disability, encompassing not only visual impairment and motor dysfunction but also symptoms such as sensory disturbances, cognitive deficits, and autonomic dysfunction. In one study of untreated NMOSD patients, it was found that 50% required wheelchairs, 50% were blind in at least one eye, and 33% had died within 5 years of their first attack (2). The identification of aquaporin-4 antibodies (AQP4^+^) as a precise diagnostic biomarker for NMOSD has advanced our knowledge of its disease pathophysiology, underscoring the pathogenesis of autoimmunity against the AQP4 water channel protein present on astrocytes in the central nervous system (3).

Over the past decade, the management of NMOSD has evolved significantly with the advent of targeted immunotherapies aimed at reducing the frequency and severity of attacks. Among several off-label therapies for NMOSD, rituximab is widely used as a therapeutic intervention as a chimeric monoclonal antibody that targets CD20-positive B-cells (4). However, not all patients respond adequately to rituximab, and some may experience breakthrough attacks, adverse events, develop resistance, or encounter reimbursement challenges (5).

Inebilizumab, a humanized and glycoengineered monoclonal antibody targeting CD19-positive B-cells, was approved for the treatment of AQP4^+^ NMOSD based on the results of the N-MOmentum (NCT02200770) trial (6). As the largest global study ever conducted for NMOSD, this double-blind, placebo-controlled, randomized phase III trial enrolled 230 patients across multiple international sites to assess the efficacy and safety of inebilizumab in NMOSD patients.

With a primary endpoint focused on assessing the time to an adjudicated NMOSD attack, and secondary endpoints centered on disability progression, hospitalization rate, and quality of life, the randomized control period (RCP) of the N-MOmentum trial demonstrated a 77% reduction in the risk of attacks compared to placebo in the first 6.5 months. Notably, enrollment in the trial was halted prematurely based on the recommendation of the data-monitoring committee, which, recognizing clear evidence of efficacy, advised the early cessation of the study due to a conditional power exceeding 99% (6). The long-term data of the optional open label extension period (OLP) demonstrated a 97% attack risk reduction in patients that received ≥2.5 years of inebilizumab treatment compared to the placebo group in the RCP (6). An additional *post-hoc* analysis assessed the efficacy and safety in a small cohort of patients (*n* = 17/230) who were previously exposed to rituximab prior to initiating inebilizumab, with seven out of 17 having recorded breakthrough NMOSD attacks despite rituximab treatment, and four out of those seven experiencing more than one attack during their rituximab treatment course (7). The annualized attack rate of this cohort of participants with prior rituximab use decreased from 0.78 at baseline to 0.08 with inebilizumab treatment and was similar to the annualized attack rate of participants without prior rituximab use (0.10). Furthermore, none of the seven participants in the study who experienced breakthrough attacks while previously being treated with rituximab went on to experience an attack while taking inebilizumab. Inebilizumab may be effective in preventing attacks in NMOSD patients regardless of prior rituximab experience, and patients who experienced treatment failure or sub-optimal treatment with rituximab may still experience significant clinical benefits after initiating inebilizumab treatment (7). However, limited real-world data exist on the efficacy and safety of inebilizumab, particularly in patients who have transitioned from other treatments, such as rituximab.

This retrospective analysis evaluates the characteristics of patients who have transitioned from rituximab to inebilizumab and assesses the clinical changes that occur during and after the transition in a cohort of 14 NMOSD patients. We also examine the diagnosis, referral pathway, and medical history of these patients to better understand the potential characteristics of viable transition candidates in real-world utilization. Through this retrospective case series, our objective is to identify the drivers for switching from rituximab to inebilizumab treatment, to provide insights into the effectiveness and safety of inebilizumab in a real-world clinical setting, and to evaluate inebilizumab as a therapeutic alternative for NMOSD patients who do not adequately respond to or experience adverse events with rituximab.

## 2 Case presentation

A retrospective study of patients with NMOSD who had transitioned from rituximab to inebilizumab was designed. De-identified patient data including demographics (e.g., age, gender, and diagnoses), disease characteristics, referral pathways, treatment history, drivers of treatment decision making, and safety and efficacy outcomes were collected. The original case intake form is available (Supplementary material S1). Supporting magnetic resonance imaging (MRI) were aggregated for analysis. The study population was drawn from seven institutions in the United States (Supplementary material S2). A total of 14 patients met the inclusion criteria of being 18 years of age or older and having a diagnosis of NMOSD with a transition from rituximab to inebilizumab treatment (either immediately or after a treatment gap) with at least one dose of administered inebilizumab.

Permissible conditions included patients who were on a combination of rituximab and other treatments [e.g., mycophenolate mofetil (MMF)] prior to transitioning to inebilizumab, and treatment gaps between rituximab and inebilizumab were permissible provided no *new* long-term immunosuppressants or biologics were introduced during the gap. Any dosing intervals extending beyond 6 months after rituximab cessation were documented but did not lead to exclusion. In addition, AQP4 antibody status was not a factor for exclusion. Patients who, *after* discontinuing rituximab, started and remained on other long-term immunosuppressants [e.g., azathioprine (AZA), MMF] or biologics (e.g., eculizumab) before starting inebilizumab were excluded from further analysis.

Descriptive statistics summarized patient characteristics and clinical outcomes. Continuous variables were reported as means, while categorical variables were reported as frequencies and percentages. Outcome measures included changes in NMOSD attack rate, expanded disability status scale (EDSS) score, and transition-related adverse events. Clinical evaluation, patient medical history, clinical course, patient symptoms/neurological deficits, treatment and monitoring with rituximab or inebilizumab, and patient-reported outcomes and clinical observations were all recorded for each case report.

### 2.1 Patient demographics

Of the 14 patients examined, the majority identified as female (78.6%, *n* = 11) and Black or African American (57.1%, *n* = 8). Two (14.3%) reported to be of Hispanic or Latino ethnicity. The mean age at the onset of the first NMOSD symptom was 37.8 years, ranging between 18 and 62 years; however, details for four patients remain undisclosed (Table 1).

**Table 1:** Patient demographics, journey to diagnosis, clinical evaluation, and medical history; *N* = 14.

Demographics		Patients *n* (%)
Gender	Female	11 (78.6%)
	Male	1 (7.1%)
	N/A (not available)	2 (14.3%)
Age at first symptom onset	Range: 18–62 years old	10 (71.4%)
	Mean: 37.8 years	4 (28.6%)
	N/A (not available)	
Race	Black or African American	8 (57.1%)
	White	5 (35.7%)
	Other	1 (7.1%)
Residence (state)	Maine (ME)	1 (7.1%)
	Kentucky (KY)	1 (7.1%)
	Georgia (GA)	1 (7.1%)
	Washington D.C. (D.C.)	3 (21.4%)
	New York (NY)	4 (28.6%)
	Illinois (IL)	2 (14.3%)
	Virginia (VA)	2 (14.3%)
Occupation	Retired/Disability	1 (7.1%)
	Nursing/Disability	1 (7.1%)
	Retail/Cashier	1 (7.1%)
	Unemployed	1 (7.1%)
	N/A (not available)	10 (71.4%)
Journey to NMOSD diagnosis		Patients *n* (%)
Referred to treating HCP	Referred	12 (85.7%)
	Referral sources:	
	General Neurologists	6 (42.8%)
	Primary Care Physicians	2 (14.3%)
	Emergency Rooms	3 (21.4%)
	Neuro-ophthalmologists	1 (7.1%)
	N/A (not available)	2 (14.3%)
Patient already diagnosed upon referral	Yes	11 (78.6%)
	General Neurologist	7 (50.0%)
	Ophthalmologist	1 (7.1%)
	Neuro-ophthalmologist	1 (7.1%)
	Neuro-oncologist	1 (7.1%)
	MS Specialist	1 (7.1%)
	No	1 (7.1%)
	N/A (not available)	2 (14.3%)
Referred for a second opinion	Referred	5 (35.7%)
	Not referred	7 (50.0%)
	N/A (not available)	2 (14.3%)
Treatment status at the time of referral	Already receiving treatment	7 (50.0%)
	Not receiving treatment	7 (50.0%)
Continuity of care	Patients that have their case report-submitting physician continuing as their current treating physician	14 (100%)
Clinical evaluation		Patients *n* (%)
AQP4 seropositivity	Seropositive	13 (92.9%)
	Seronegative	1 (7.1%)
Serologic testing dates	Ranged from 01/01/2011 to 12/20/2022	9 (64.3%)
	Not available	5 (35.7%)
AQP4 test method	Cell based assay (CBA)	11 (78.6%)
	ELISA and CBA	1 (7.1%)
	ELISA	1 (7.1%)
	Not available	1 (7.1%)
MOG testing results	Seronegative/indeterminate/low titer	1 (7.1%)
	Not available	13 (92.9%)
NMOSD diagnoses	Occurred between 01/01/2011 and 11/01/2021	14 (100%)
Core clinical characteristics	Optic neuritis (ON)	6 (42.9%)
	Transverse myelitis (TM)	10 (71.4%)
	TM involving ≥3 vertebral segments	8 (80.0%)
	Area postrema Syndrome (APS)	2 (14.3%)
	Acute brainstem syndrome	1 (7.1%)
Imaging files taken	Yes	11 (78.6%)
	No	3 (21.4%)
Medical history (Prior to transitioning to rituximab)		Patients *n* (%)
Time from symptom onset to diagnosis	Mean: 28.8 months (2.4 years)	14 (100%)
	Range: 0–122 months (0–10.2 years)	
Autoimmune comorbidities	Hashimoto’s thyroiditis disease	1 (7.1%)
	Systemic lupus erythematosus	1 (7.1%)
Previous NMOSD treatment history	No previous treatment	5 (35.7%)
	Previously treated with Steroid, PLEX, or IVIG	7 (50.0%)
	Previously treated with oral ISTs (AZA or MMF)	7 (50.0%)
	CD20 Agent	1 (7.1%)
First-line treatment (Mean duration: 2.9 years)	Intravenous Methylprednisolone	3 (21.4%)
	Azathioprine (AZA)	2 (14.3%)
	Mycophenolate Mofetil (MMF)	2 (14.3%)
	Intravenous immunoglobulin (IVIG)	1 (7.1%)
	Plasma exchange (PLEX)	1 (7.1%)
Second-line treatment (Mean duration: 3.22 years; 0–9.54 years)	Prednisone	3 (21.4%)
	Plasma exchange (PLEX)	1 (7.1%)
	Mycophenolate Mofetil (MMF)	1 (7.1%)
Reasons for discontinuation of treatment	Breakthrough disease	5 (35.7%)
	Worsening symptoms	2 (14.3%)
	Insurance	1 (7.1%)
Attacks between 2008 and 2022	Total number of attacks	31 (100%)
	Optic neuritis (ON)	7 (22.6%)
	Transverse myelitis (TM)	11 (35.5%)
	ON and TM	1 (7.1%)
	Area postrema syndrome (APS)	1 (7.1%)
	APS combined with TM	7 (22.6%)
	Other (encephalopathy, ataxia, brainstem syndrome, and lower extremity leg weakness)	4 (12.9%)
Patient symptoms/neurological deficits; *N* = 14		
Symptoms	Category	Patients *n* (%)
Visual Acuity (OD, right eye)	Visual	5 (35.7%)
Visual Acuity (OS, left eye)	Visual	2 (14.3%)
Double vision	Visual	1 (7.1%)
Neuropathic pain	Motor/Sensory	9 (64.3%)
Gait impairment	Motor/Sensory	11 (78.6%)
Numbness	Motor/Sensory	4 (28.6%)
Paresis/Paralysis	Motor/Sensory	6 (42.9%)
Spasticity	Motor/Sensory	4 (28.6%)
Spasms	Motor/Sensory	4 (28.6%)
Fatigue	Other	6 (42.9%)
Headache	Other	4 (28.6%)
Depression	Other	3 (21.4%)
Anxiety	Other	2 (14.3%)
Speech deficiencies	Other	1 (7.1%)
Loss of coordination	Other	1 (7.1%)
Loss bowel/bladder control	Other	3 (21.4%)
Brain fog	Other	2 (14.3%)
Seizure	Other	1 (7.1%)
Difficulty swallowing	Other	1 (7.1%)
Vitamin B12 deficiency	Other	1 (7.1%)
Mood changes^*^	Other	1 (7.1%)

^*^Mood changes refers to a case of paranoid delusional disorder confirmed by psychiatry. n, number; %, percentage; HCP, Health care professional; NMOSD, Neuromyelitis optica spectrum disorder; AQP4, Aquaporin 4; MOG, Myelin oligodendrocyte glycoprotein; ELISA, Enzyme-linked immunosorbent assay; CBA, Cell-based assay; ON, Optic neuritis; TM, Transverse myelitis; PLEX, Plasma exchange; IVIG, Intravenous immunoglobulin; ISTs, Immunosuppressants; AZA, Azathioprine; MMF, Mycophenolate mofetil.

### 2.2 Referral patterns

Referrals to the treating physician were predominant (85.7%) and primarily from general neurologists (42.8%, *n* = 6) and emergency room visits (21.4%, *n* = 3). Half of the patients (50%, *n* = 7) were already receiving treatment at the time of referral (Table 1).

### 2.3 Clinical characteristics

Among the patients, 92.9% (*n* = 13) were AQP4 seropositive. While 78.6% (*n* = 11) were diagnosed using a cell-based assay (CBA), one patient (7.1%, *n* = 1) was identified as seropositive through the enzyme-linked immunosorbent assay (ELISA). The remaining patient, identified as AQP4 seronegative with indeterminate myelin oligodendrocyte glycoprotein (MOG) test results, met the criteria outlined by the International Panel for Neuromyelitis Optica Diagnosis (IPND) for seronegative NMOSD, and the physician made the decision to treat the patient off-label with inebilizumab. This diversity in serological profiles underscores the nuanced diagnostic landscape within the study cohort (Table 1).

Diagnoses were made between January 2011 and November 2021. Optic neuritis (ON) and transverse myelitis (TM) emerged as the leading clinical features, present in 42.9% (*n* = 6) and 71.4% (*n* = 10) of patients, respectively. Among TM diagnoses, more than three vertebral segments were involved in 80.0% (*n* = 8). Cases also included Area Postrema Syndrome (APS) in 14.3% (*n* = 2) and Acute Brainstem Syndrome in 7.1% (*n* = 1, Table 1).

### 2.4 Historical treatment (excluding rituximab)

From symptom onset, the average time to NMOSD diagnosis was 2.4 years (28.8 months), with some diagnosed at time of first attack and others taking up to 10.2 years (ranging from 0 to 122 months). Notable autoimmune comorbidities were found in two patients: Hashimoto’s thyroiditis (*n* = 1) and systemic lupus erythematosus (*n* = 1). Other patients had histories of varied treatments, including corticosteroids, plasma exchange (PLEX), intravenous immunoglobulin (IVIG) (*n* = 7), oral immunosuppressants such as AZA or MMF (*n* = 7), and one with a prior CD20 therapy (*n* = 1, Table 1).

Between 2008 and 2022, the cohort recorded a total of 31 attacks. These comprised ON only (*n* = 7), TM only (*n* = 11), both ON and TM (*n* = 7), APS (*n* = 1), APS combined with TM (*n* = 1), and other manifestations like seizure, encephalopathy, ataxia, brainstem syndrome, and lower extremity leg weakness (*n* = 4, Table 1). The manifestation of lower extremity leg weakness was distinct and not attributed to transverse myelitis (TM).

### 2.5 Treatment and monitoring with rituximab

Five patients were treatment naïve prior to initiating rituximab. For those on prior therapy (*n* = 9), the average time from diagnosis to first rituximab infusion was 23.6 months. The average duration of rituximab treatment was 38.6 months (approximately 3.2 years), with individual durations spanning from 3 to 84 months (0.3–7 years). All patients (*n* = 14) underwent rituximab infusions biannually at a dosage of 1,000 mg every 6 months (Table 2).

**Table 2:** NMOSD patients on rituximab: clinical outcomes and treatment experiences; *N* = 14.

Clinical outcomes and treatment experiences		
Category	Details/Notes	Patients *n* (%)
Average treatment duration (months)	Mean: 38.6 months (3.2 years)	14 (100%)
	Range: 3 months–84 months (0.3–7 years)	
Infusion reactions	Encephalopathy, brain fog, headache, itchy throat, itchy left face	2 (14.3%)
Dosing changes due to incomplete B-cell depletion	Yes	0 (0%)
	No	14 (100%)
Infections	None	12 (85.7%)
	Unknown	1 (7.1%)
	Mild urinary tract infections	1 (7.1%)
Rituximab + Other IST	No	12 (85.7%)
	IVIG for low antibody levels	1 (7.1%)
	Mycophenolate mofetil (MMF)	1 (7.1%)
Concurrent steroid use	Yes	4 (28.6%)
	Treatment duration <4 weeks	3 (75.0%)
	Treatment duration >12 weeks	1 (25.0%)
Attacks	Reported patients	10 (71.4%)
	Total attacks across reported patients	17
	Reported causes:	
	Breakthrough disease	10 (58.8%)
	Treatment delay	7 (41.2%)
Catalyst for transitioning to inebilizumab	Breakthrough disease	6 (42.9%)
	Patient preference	7 (50.0%)
	Requested home infusion	1 (7.1%)
	FDA approved and superior clinical efficacy	6 (42.9%)
	Treatment delays	1 (7.1%)

n, number; %, percentage; OS, Oculus sinister-left eye; OD, Oculus dexter-right eye; Min, Minimum; Max, Maximum; IST, Immunosuppressant; IVIG, Intravenous immunoglobulin; MMF, Mycophenolate mofetil.

Two patients received rituximab in conjunction with other immunosuppressive therapies, which included IVIG due to hypogammaglobulinemia (*n* = 1) or MMF (*n* = 1). Corticosteroids were utilized concurrently by 28.6% (*n* = 4) of the cohort, with durations spanning from less than 4 weeks (*n* = 3) to over 12 weeks (*n* = 1, Table 2).

Throughout the rituximab treatment period, 17 total attacks were documented in 71.4% (*n* = 10) of the patients. These attacks were primarily attributed to breakthrough disease (*n* = 10) and treatment delays (*n* = 7, Table 2). Across the six patients who experienced breakthrough disease on rituximab treatment, the average duration of rituximab treatment at the time of any NMOSD attack was about 26.5 months. However, when focusing on the final attack only (considering patients may have experienced more than one attack), the average duration was approximately 22.5 months. The average duration of treatment delay with rituximab was approximately 48 months among the four patients included in the analysis. In total, seven treatment delays were recorded, with an average treatment delay of 27 months (range: 2–55 months) across all patients. Breakthrough disease events and treatment delays are elaborated upon in Supplementary material S3, S4.

Overall, two patients were reported to have experienced rituximab infusion-related reactions despite the administration of pre-infusion medications, which included symptoms such as encephalopathy, brain fog, headache, itchy throat, and itchy face. Infections were reported in one patient, specifically mild urinary tract infections, which were resolved with appropriate antibiotic treatment.

Monitoring of patients involved routine assessments of IgG, IgM, and B-cell depletion, complemented by occasional evaluations like complete blood count (CBC) and a comprehensive metabolic panel (CMP). The frequency of these tests varied, ranging from an “as-needed” basis to annual assessments.

**Figure 1:**
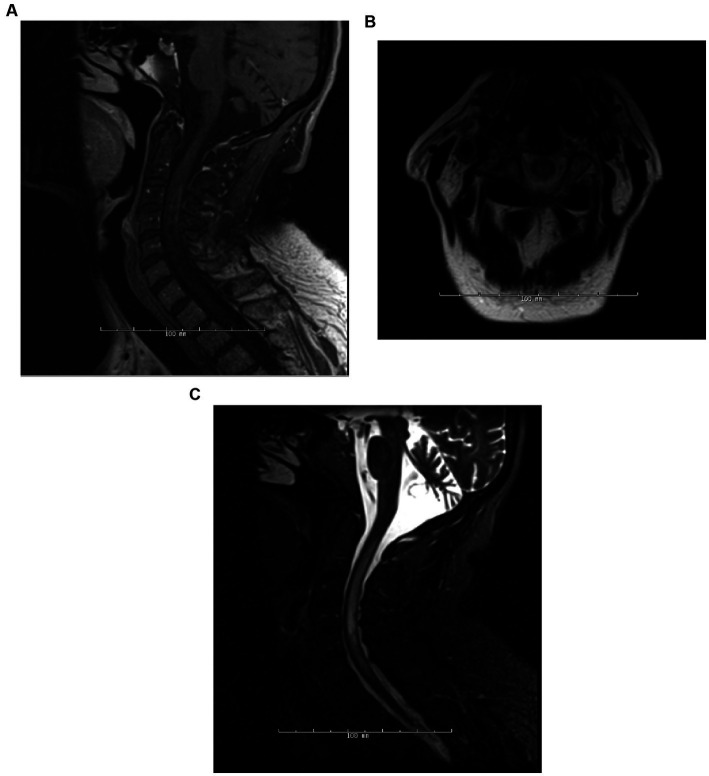
Patient 5 (*detailed under* “*breakthrough disease*” in Supplementary material S3), 6 months post-initiation of rituximab treatment, the patient manifested an attack, encompassing both TM and encephalopathy, with a concurrent CD19+ B-cell count of 3%. MRI assessments during this episode included Sagittal T1-post contrast **(A)**, Axial T2 **(B)**, and Sagittal STIR **(C)** of the spine.

Imaging techniques, including MRI of the brain, spine, optic orbits, and computed tomography (CT) scans of the spine and brain, were employed to track disease progression and evaluate treatment efficacy (Figure 1). The frequency of these imaging assessments was patient-specific, occurring either annually, biannually, or as needed.

Clinical symptomatology was monitored, encompassing motor, visual, and sensory functions, and specific symptoms like leg spasms, clonus, hemiparesis, numbness, burning sensations, and vision complications. Some patients underwent additional ophthalmological exams and standard neurological evaluations, including the Timed 25-Foot Walk (T25-FW) and the 9-Hole Peg Test.

Lastly, adverse events such as hypogammaglobulinemia, infusion-related reactions, coagulopathies, and infections were monitored. The monitoring frequencies for these adverse events were patient-specific, with intervals ranging from quarterly to annual assessments.

### 2.6 Treatment and monitoring with inebilizumab

Among the 14 patients transitioning from rituximab to inebilizumab, 42.9% (*n* = 6) did so due to breakthrough disease. For 50.0% (*n* = 7), personal preference was the deciding factor, with one patient specifically valuing inebilizumab for its home infusion option. Among these, the Federal Drug Administration (FDA) approval of inebilizumab for AQP4^+^ NMOSD influenced the decision for 42.9% (*n* = 6). Treatment delay with rituximab prompted the change to inebilizumab for one patient (Table 2).

When transitioning from rituximab to inebilizumab, 21.4% (*n* = 3) did so within 3 months, 50.0% (*n* = 7) transitioned between 4 and 6 months, and 28.6% (*n* = 4) transitioned after 6 months relative to their prior rituximab infusion. For inebilizumab loading doses, four patients (28.6%) received one dose while 10 patients (71.4%) received two doses (Table 3). For context, the dosing regimen in the N-MOmentum trial prescribed a 300 mg IV dose on day 1 and another on day 15 as part of the loading phase, followed by 300 mg maintenance doses every 6 months thereafter. On average, inebilizumab treatment duration spanned approximately 19.3 months, with a median of 19 months and individual treatment durations ranging from 9 to 35 months.

**Table 3:** NMOSD patients on inebilizumab: treatment and monitoring, patient reported outcomes, and clinical observations; *N* = 14.

Treatment and monitoring		
Category	Subcategory	Patients
		*n* (%)
Transition period: time from last rituximab dose to first inebilizumab dose (months)	< 3 months	3 (21.4%)
	4–6 months	7 (50.0%)
	>6 months	4 (28.6%)
Other treatment between transition?	Yes—MMF	1 (7.1%)
	No	13 (92.9%)
Number of loading doses	One loading dose	4 (28.6%)
	Two loading doses	10 (71.4%)
Duration of inebilizumab use	< 6 months	0 (0.0%)
	6–12 months	1 (7.1%)
	13–24 months	11 (78.6%)
	> 24 months	2 (14.3%)
Infusion reactions	Yes	0 (0.0%)
	No	14 (100.0%)
Combined with immunosuppressants?	Yes—MMF	1 (7.1%)
	No	13 (92.9%)
Concurrent steroid use (including corticosteroid taper)?	Yes	0 (0.0%)
	No	14 (100.0%)
Frequency of lab assessments (IgG, IgM, and B-cell counts)	Once to date	1 (7.1%)
	Every 6 months	4 (28.6%)
	Every 6–12 months	3 (21.4%)
	Unknown	6 (42.9%)
CD19+ B cell levels	Negligible/Zero	14 (100.0%)
Frequency of imaging (MRI of brain, thoracic, cervical, and orbits)	As needed	1 (7.1%)
	Once to date	4 (28.6%)
	Every 6 months	1 (7.1%)
	Every 12 months	1 (7.1%)
	Unknown	7 (50.0%)
Frequency of adverse event evaluations (hypogammaglobulinemia, infusion-related reactions, infections, and coagulopathies)	Every infusion	1 (7.1%)
	Every 6 months	2 (14.3%)
	Every 8–9 months	1 (7.1%)
	Not mentioned/unknown	10 (71.4%)
Attacks while on inebilizumab treatment	Yes	0 (0.0%)
	No	14 (100.0%)
Chronic treatment adjustments made	Yes	0 (0.0%)
	No	14 (100.0%)
Patient reported outcomes and clinical observations; *N* = 14		
Category	Description	Patients*n* (%)
Patients reporting NMOSD changes	Reported patients	7 (50.0%)
	Improvement of symptoms^a^	6 (42.9%)
	Worsening of symptoms^b^	1 (7.1%)
Disability scales measured	EDSS	3 (21.4%)
	T25-FW	2 (14.3%)
	SF-36	2 (14.3%)
Patients with improved disability	EDSS	3 (21.4%)
	T25-FW	1 (7.1%)
	SF-36	0 (0.0%)
Patients with minimal/uncertain disability change	T25-FW	1 (7.1%)
	SF-36	2 (14.3%)
Activities of daily living/Quality of life (ADL/QOL) outcomes measured	Reported patients	10 (71.4%)
	Positive trends	6 (42.9%)
	Uncertain trends	4 (28.6%)
Improvements in symptoms^a^	Gait symptoms	4 (28.6%)
	General comfort and no new weakness	1 (7.1%)
	Upper extremity function	1 (7.1%)
Worsening symptoms^b^	Neuropathic pain, burning, spasticity, and sleepiness	1 (7.1%)

^a^Improvements in symptoms; Patient 1: saw mild improvement of gait symptoms after 5 months of inebilizumab exposure (EDSS baseline: 6.5, follow up: 6). Patient 2: saw mild improvement of gait symptoms after 7 months of inebilizumab exposure (EDSS baseline; 6, follow up 5.5). Patient 3: saw mild improvement of gait symptoms after 11 months of inebilizumab exposure (EDSS baseline: 6.5, follow up 6). Patient 4: noted improved symptomatology after 9 months of inebilizumab exposure—general feeling well, no new weakness. Patient is pleased with being on it and noted more comfortable than being on RTX. Patient 5: noted improved function in upper extremities after 4 months of inebilizumab exposure. Patient 6: noted improvement of gait symptoms after 20 months of inebilizumab exposure [T25-FW; Baseline: 33.7; Follow Up: 6.7 (11/2022)]. ^b^Worsening symptoms; despite a slight improvement recorded in SF-36 (baseline: 6; follow up: 5) after 12 months of inebilizumab exposure, one patient case reported subjective worsening of symptoms including neuropathic pain, burning, spasticity, and sleepiness. Of note, the patient was diagnosed with seronegative NMOSD (the only seronegative participant in this case series) and has been reported to include the following medical history: stroke, CKD, migraines, Hepatitis B, depression, anxiety, obesity (gastric bypass), MGUS, hypothyroidism (thyroidectomy 2000), COPD, and myelodysplastic syndrome. n, number; %, percentage; NMOSD, Neuromyelitis optica spectrum disorder; EDSS, Expanded disability status scale; T25-FW, Timed 25-foot walk test; SF-36, 36-item short-form health survey; ADL, Activities of daily living; and QOL, Quality of life.

Apart from the pre-infusion steroid administration, there were no reports of concurrent corticosteroid administration or corticosteroid tapering during the initiation of the inebilizumab loading phase. Among the 14 participants, no infusion-related reactions or infections were reported. Notably, one patient continued with MMF during the transition (but discontinued after the second dose of inebilizumab, Supplementary material S4).

Laboratory assessments were performed on various immunological markers, with IgG, IgM, and B-cells being the most common. Assessments were conducted either once to date, every 6 months (most common), or every 6 months to 1 year. All of the patients (100%, *n* = 4) who tested for B-cell enumeration reported undetectable levels of CD19+ B-cells (Table 3).

During treatment, neurological symptoms were evaluated such as visual acuity, eye pain, headaches, weakness, numbness, leg spasms, clonus, left-side weakness, burning sensations, vision issues, hemiparesis, tongue paralysis, and dysphagia. Diagnostic tests including the T25-FW and the 9-Hole Peg Test were administered to assess neurological function and mobility. Additionally, imaging studies, predominantly MRI scans of the brain and thoracic regions, were conducted using varied protocols to evaluate structural changes and abnormalities. The frequency of imaging ranged from once to date to once annually, with some patients receiving imaging on an as-needed basis.

Adverse events monitored during treatment included infusion reactions, opportunistic infections, hypogammaglobulinemia, and coagulopathies. The frequency of adverse event evaluations varied but were reported to be with each infusion (*n* = 1), every 6 months (*n* = 2), or every 8–9 months (*n* = 1). For 10 patients, adverse event information was either not evaluated or unavailable at the time of the survey, as determined from the retrospective chart review (Table 3).

### 2.7 Patient reported outcomes and clinical observations with inebilizumab

Upon evaluating 14 NMOSD patients who transitioned to inebilizumab, we found that none experienced attacks during the inebilizumab treatment phase. Additionally, there were no reported modifications to ongoing treatments after the initiation of inebilizumab.

To highlight the therapeutic impact of inebilizumab, we systematically documented relevant clinical observations. Regarding disability assessments, three patients (21.4%) underwent evaluations using EDSS, and all three documented a 0.5-point improvement. Evaluations using the T25-FW and the 36-Item Short Form Survey (SF-36) scales, were each conducted for two patients (14.3%) with varied results. A notable case involved a patient who exhibited a pronounced improvement in the T25-FW assessment, while two others showed minimal or indeterminate changes on the SF-36 scale. Moreover, a majority of the cohort (71.4%, *n* = 10) were assessed for Activities of Daily Living and Quality of Life (ADL/QOL). Of these, six patients (60.0%) exhibited improved daily life quality and functionality (Table 3).

Subsequently, we documented patient-reported outcomes. Seven of the participants (50.0%) indicated alterations in their NMOSD symptoms after the transition. Six of the seven patients reported marked symptomatic improvements. For instance, one patient highlighted enhanced gait symptoms after a 5-month period, while another noted a considerable improvement in gait over 20 months. One patient (7.1%) reported exacerbated symptoms such as neuropathic pain, burning, spasticity, and sleepiness. However, their SF-36 score was improved at the 12-month mark. This patient was uniquely diagnosed with seronegative NMOSD with a complex medical history (Table 3).

## 3 Discussion

The advent of targeted therapies has significantly transformed the treatment paradigm for NMOSD. While rituximab is frequently used off-label for NMOSD, inebilizumab, which targets CD19-positive B-cells and is FDA-approved for AQP4^+^ NMOSD, has proven efficacy in reducing NMOSD attacks as evidenced in a pivotal randomized controlled trial (6). Recent research underscores the central role of CD19+ plasmablasts in neuroimmunology disorders, suggesting that their migration into the central nervous system and function as auto-antibody producers could have implications for the observed therapeutic response in NMOSD patients transitioning from rituximab to inebilizumab (8).

Genetic considerations further differentiate rituximab from inebilizumab. Specifically, rituximab-treated patients carrying the *FCGR3A-F* allele have been shown to have a heightened risk of relapse (9). In contrast, inebilizumab-treated participants in the N-MOmentum trial displayed consistent outcomes irrespective of the *FCGR3A* genotype, reinforcing the drug’s targeted efficacy (10). The absence of neutralizing anti-drug antibodies (ADA) with inebilizumab additionally offers a significant advantage, ensuring sustained drug activity and reduced immunogenic reactions.

Our comprehensive analysis of 14 patients transitioning from rituximab to inebilizumab provides valuable insights into its real-world application, efficacy, and safety. Notably, none of these patients, even those who previously had disease breakthroughs on rituximab, experienced attacks under inebilizumab, echoing findings from the N-MOmentum trial. Prior real-world studies reporting on the treatment transition to inebilizumab in NMOSD are scarce. A published retrospective study of medical records in 164 AQP4+ NMOSD patients revealed that transitions from one therapy to the next may be associated with an increased relapse rate if done for “non-medical” reasons; however, these patients had an average of approximately 3 months of washout between medications (range 1–2,810 days) (11).

As a promising and versatile therapeutic agent, the introduction of inebilizumab has shown optimistic results for NMOSD patients. Most patients reported symptom relief and stable disability scores, underscoring the need for individualized care and consistent monitoring. The preference expressed by patients toward inebilizumab underlines the importance of patient-centric care in decision making. As the NMOSD therapeutic landscape evolves, ensuring patients are well-informed and involved in treatment decisions is critical. This sentiment suggests that beyond mere clinical outcomes, factors such as administration methods, treatment frequency, side effects, and individual perceptions significantly influence treatment choices. Furthermore, our study spotlighted a pressing real-world challenge: treatment disruptions due to insurance complications. Such disruptions can have severe ramifications on patient health and can inflate healthcare costs, emphasizing the need for uninterrupted access to transformative therapies and comprehensive discussions between healthcare providers, insurers, and policymakers.

Though insightful, our study possesses limitations characteristic of retrospective analyses, such as potential selection biases and the absence of a control group. It is important to note that the dataset used is partially incomplete, which may affect the robustness of our conclusions. Additionally, the modest sample size and the relatively short follow-up duration necessitate a careful interpretation of the findings. Furthermore, monitoring of B cell depletion and quantitative serum immunoglobulin levels are important during treatment with inebilizumab and this information would have provided additional support for our findings of the response to treatment.

## 4 Conclusion

Neuromyelitis optica spectrum disorder is a debilitating neurological condition that demands effective and consistent therapeutic strategies. Our retrospective analysis highlights the potential of inebilizumab as a valuable treatment option, especially for patients who may not derive optimal outcomes with rituximab.

However, as with all therapies, individualized care is paramount. The variability in patient responses emphasizes the importance of continued monitoring and patient-centric decision-making. Furthermore, challenges such as treatment interruptions due to insurance barriers highlight the need for a more integrated approach to patient care, encompassing both clinical and socio-economic facets.

In conclusion, while inebilizumab offers potential advantages for NMOSD patients, achieving optimal patient outcomes is a multi-dimensional endeavor. This journey mandates the concerted efforts of clinicians, researchers, patients, and policymakers. The pursuit for more comprehensive data and a deeper understanding persists, all aimed at enhancing the quality of life for those impacted by NMOSD.

## Data availability statement

The datasets presented in this article are not readily available because of ethical and privacy restrictions. Requests to access the datasets should be directed to the corresponding author.

## Ethics statement

Ethical review and approval was not required for the study on human participants in accordance with the local legislation and institutional requirements. Written informed consent from the patients/participants or patients/participants’ legal guardian/next of kin was not required to participate in this study in accordance with the national legislation and the institutional requirements. Written informed consent was obtained from the individual(s) and/or minor(s)’ legal guardian/next of kin for the publication of any potentially identifiable images or data included in this article.

## Author contributions

BO: Data curation, Writing – review & editing. GR: Data curation, Writing – review & editing. JH: Data curation, Writing – review & editing. MZ: Data curation, Writing – review & editing. TD: Data curation, Writing – review & editing. TK: Data curation, Writing – review & editing. UO: Data curation, Writing – review & editing. JP: Conceptualization, Data curation, Formal Analysis, Investigation, Methodology, Project administration, Supervision, Writing – original draft, Writing – review & editing. KP: Conceptualization, Data curation, Formal Analysis, Investigation, Methodology, Project administration, Resources, Supervision, Writing – original draft, Writing – review & editing. ML: Data curation, Investigation, Writing – review & editing.
