# Supplementary material for: Case report: Transition from anti-CD20 therapy to inebilizumab for 14 cases of neuromyelitis optica spectrum disorder
Source: Front Neurol. 2024 Apr 16;15:1352779. doi: 10.3389/fneur.2024.1352779 (PMC11060151; doi:10.3389/fneur.2024.1352779)
Supplement: Supplementary file 1 [file Data_Sheet_1.docx]

Case Report: Transition from anti-CD20 therapy to inebilizumab for 14 cases of Neuromyelitis Optica Spectrum Disorder

Benjamin Osborne^1^, Gabriela Romanow^2^, Michael Hemphill^3^, Myassar Zarif^4^, Tracy DeAngelis^5^, Tyler Kaplan^6^, Unsong Oh^7^, Johnathan Pinkhasov^8^, Kristina Patterson^8^, Michael Levy^2*^

**Supplementary Material**

**S1. Neuromyelitis Optica Spectrum Disorder (NMOSD) Rituximab Transition to Inebilizumab: Case Intake Form**

**
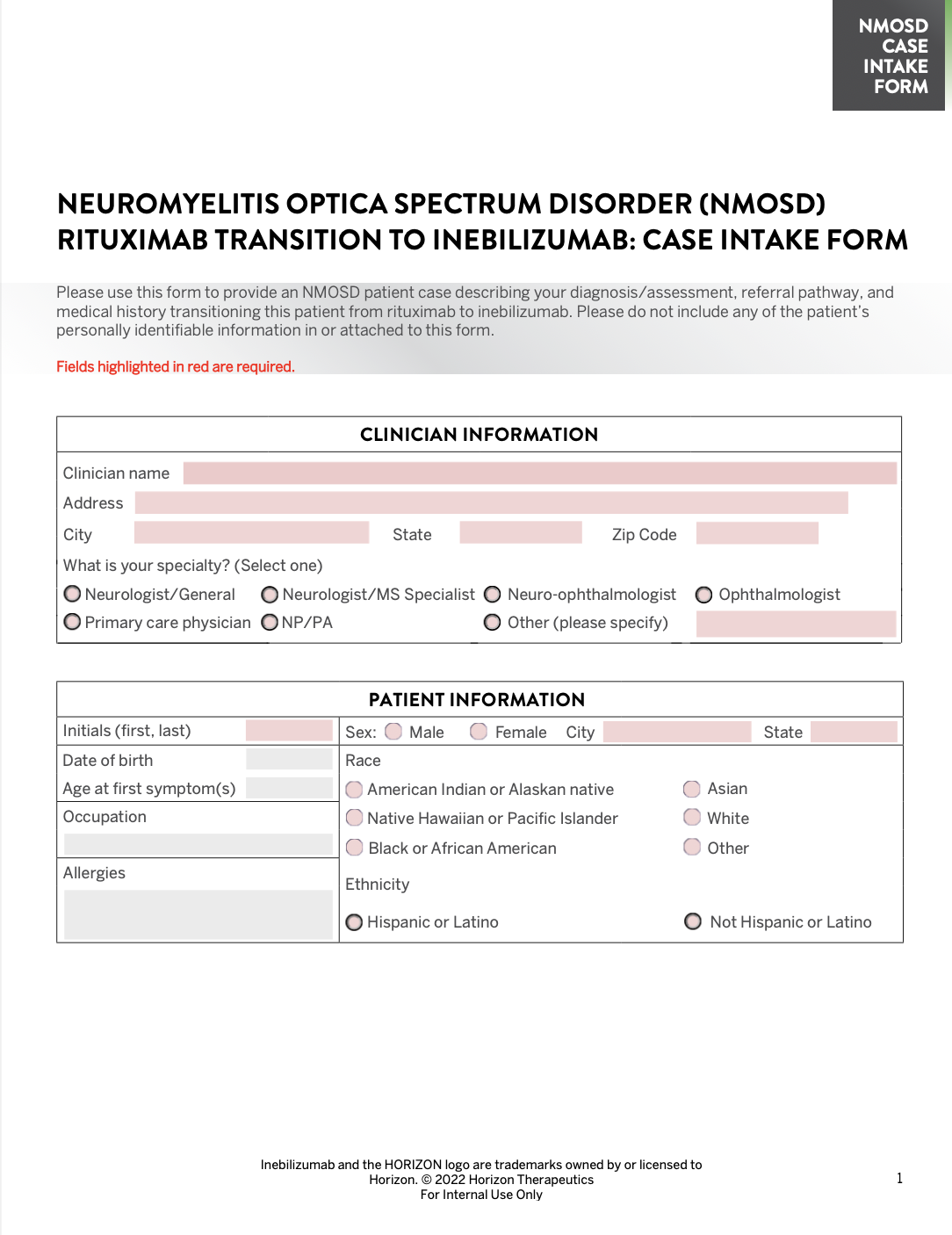
**

**
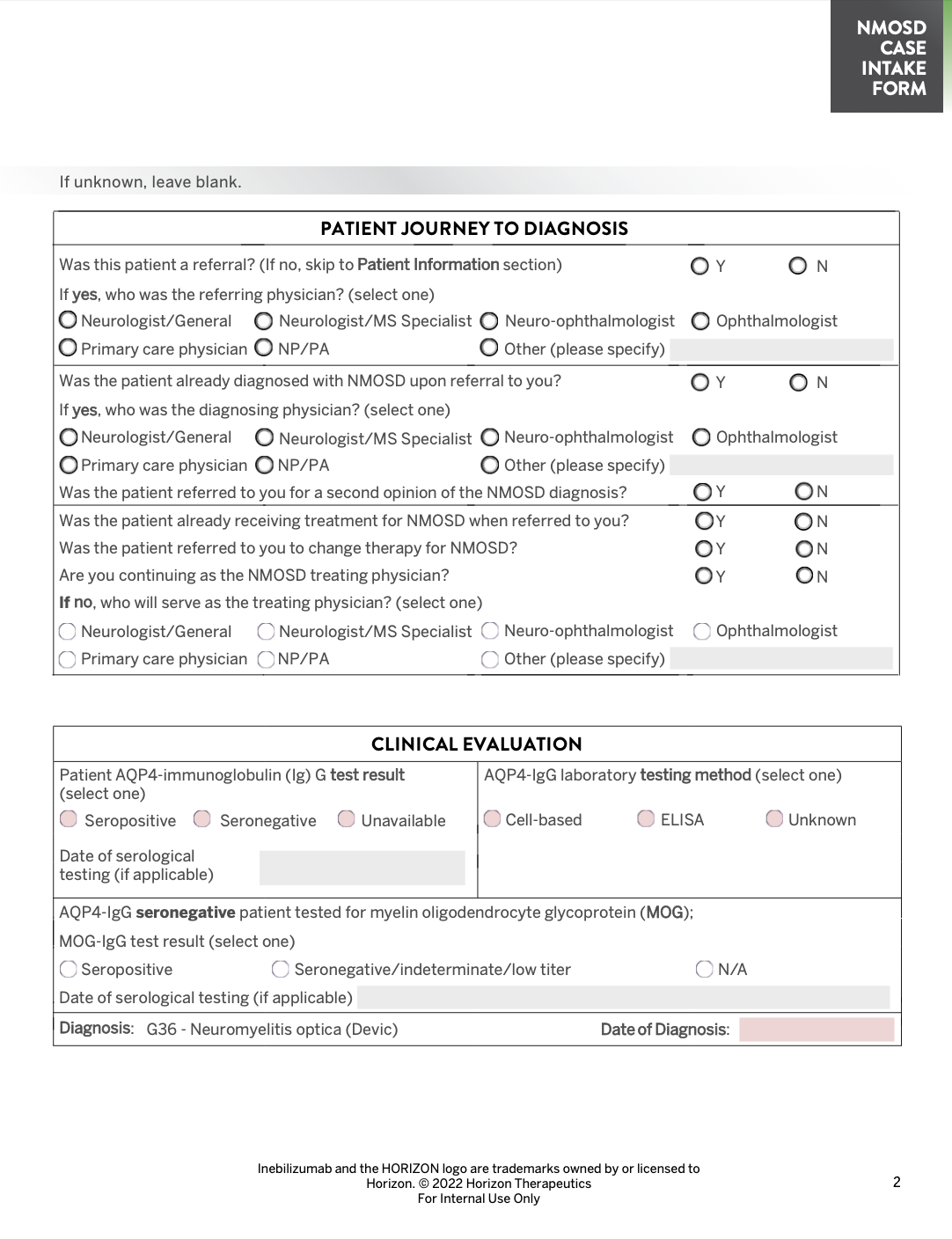
**

**
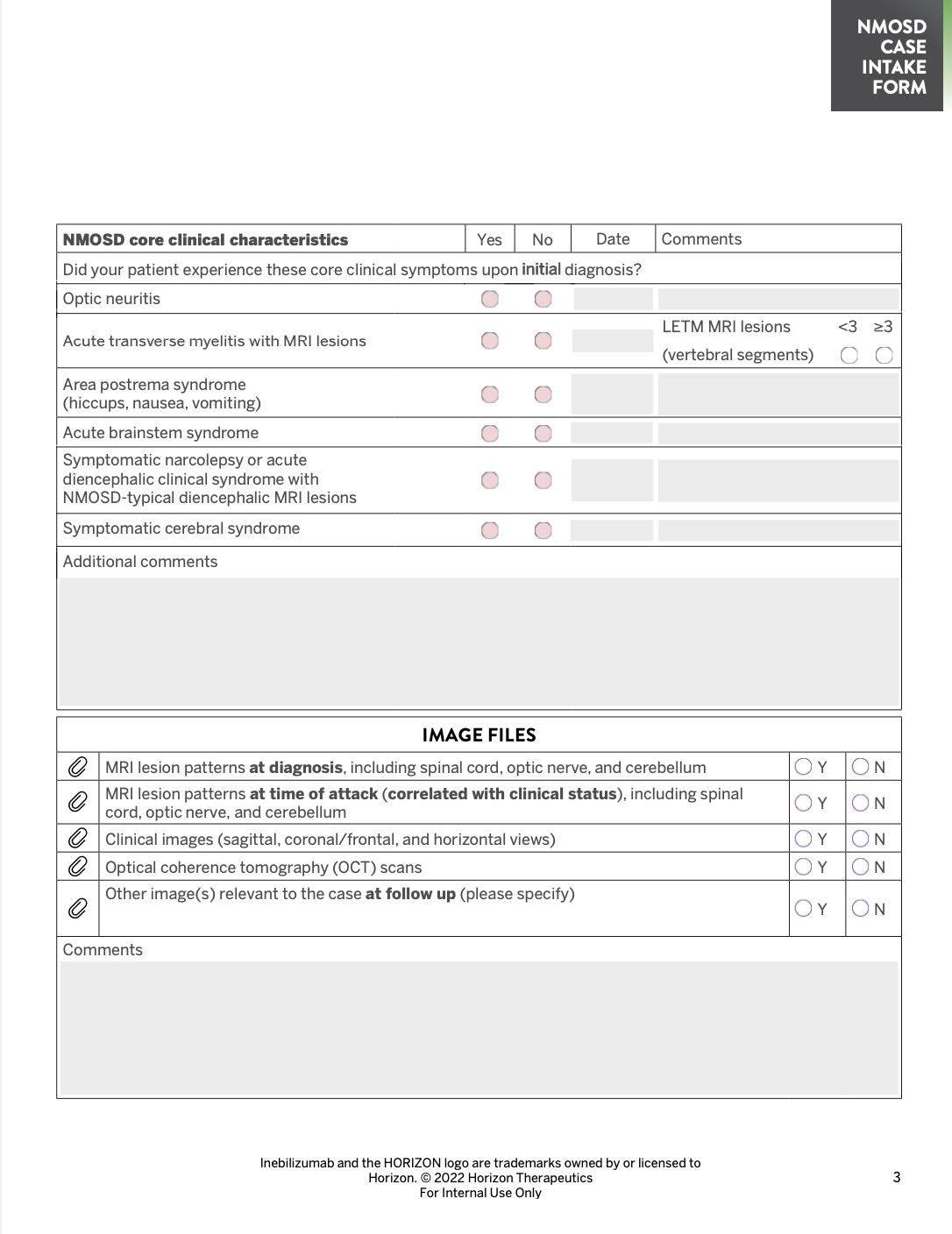
**

**
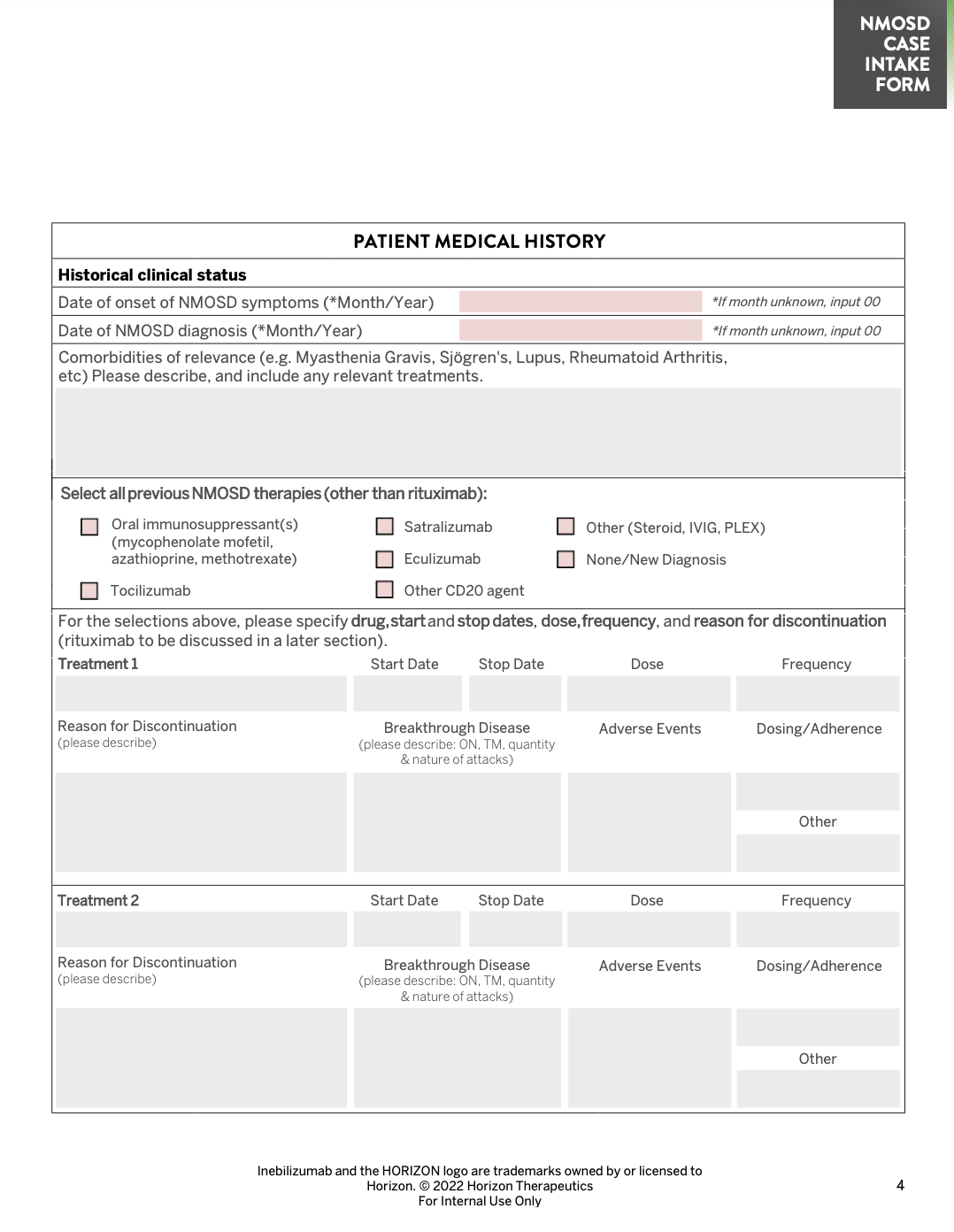
**

**
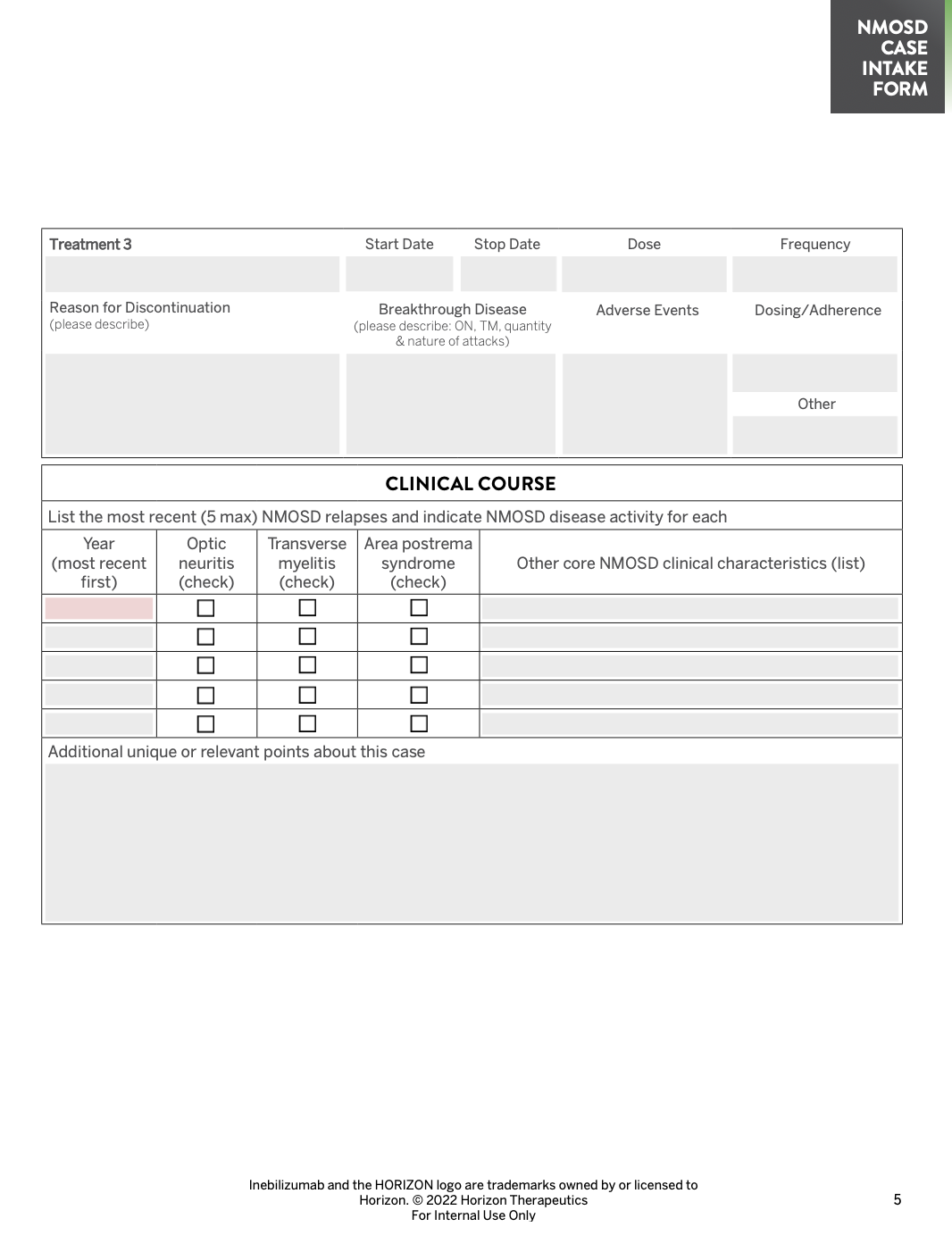
**

**
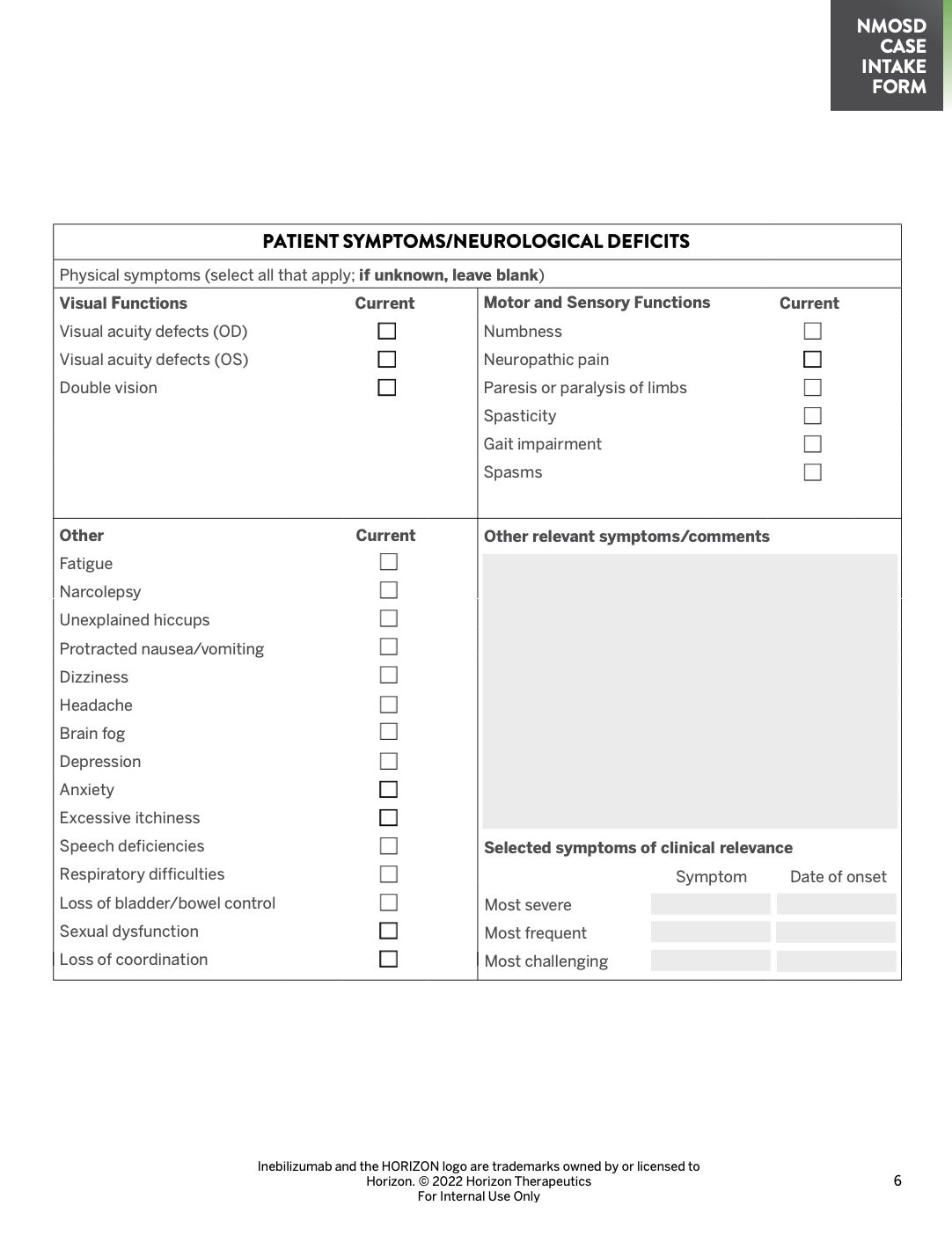
**

**
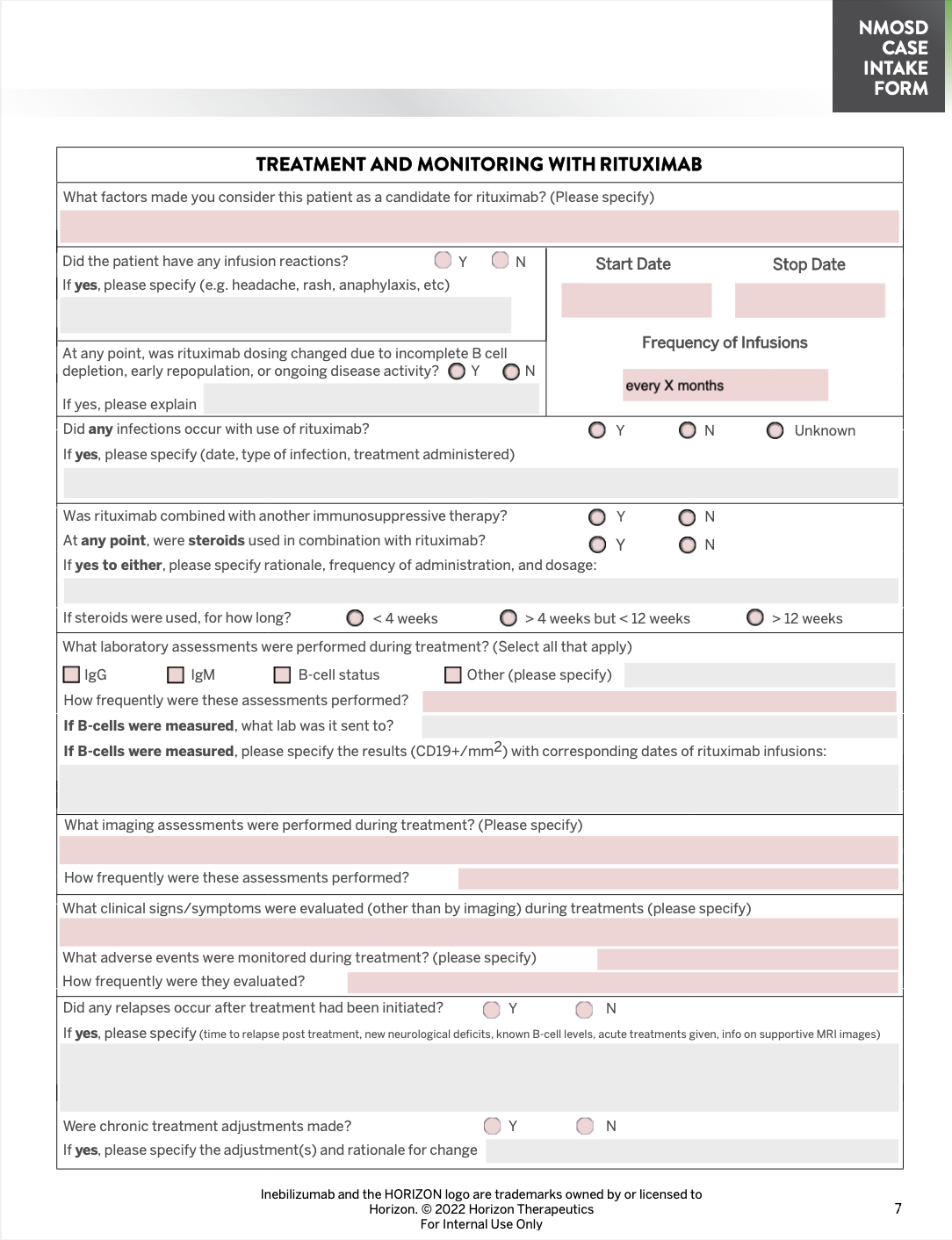
**

**
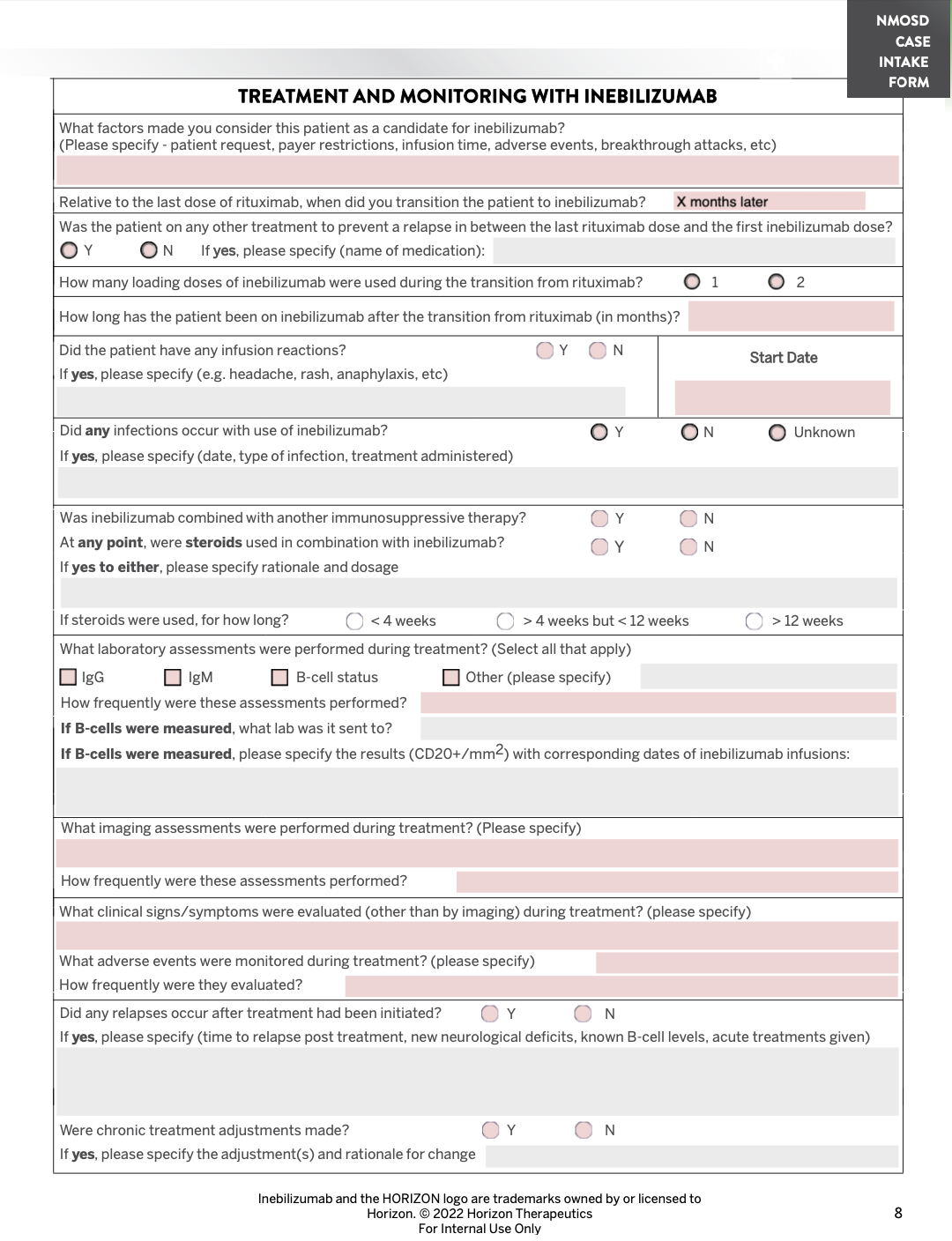
**

**
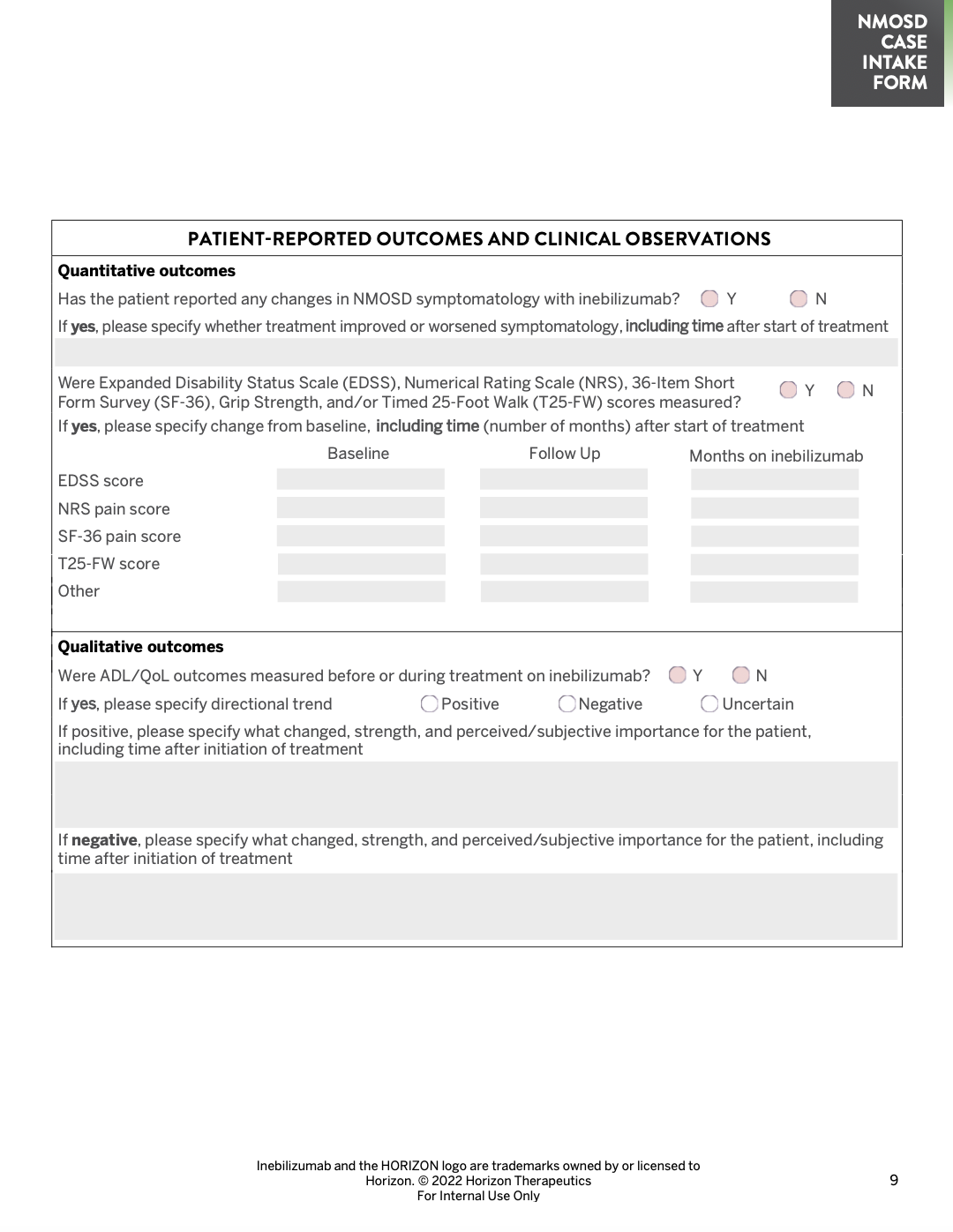
**

**S2. Institutions in the United States that Comprised the Study Population**

1. Department of Neurology, Georgetown University Medical Center, Washington, D.C. (3 patients)
2. Neurological Clinical Research Institute, Massachusetts General Hospital, Boston, MA (2 patients)
3. Savannah Neurology Specialists, Savannah, GA (1 patient)
4. South Shore Neurologic Associates, Patchogue, NY (3 patients)
5. Neurological Associates of Long Island, New Hyde Park, NY (1 patient)
6. Department of Neurology, Rush University Medical Center, Chicago, IL (2 patients)
7. Department of Neurology, Virginia Commonwealth University, Richmond, VA (2 patients)

**S3. Breakthrough Disease (attacks recorded: 10 across 6 patients)**

- Patient 1: Two attacks (both TM and ON) occurred within 3 months after initiating rituximab treatment; CD19+ B-cell count was found to be 2 cells/µL by fluorescence activated cell sorting (FACS). One year after the second attack, another attack (TM and ON) occurred and MMF 1g BID was added as combination treatment. After 1.5 years of being on combo therapy, the patient experienced another mild attack (upper extremity weakness). However, it is important to note RTX was last given 8 months prior to mild attack event, and not again till 2 months after mild attack event (biosimilar this time).
- Patient 2: Attack (ON in left eye) occurred 3 months after initiating rituximab treatment, which lasted for a duration of 2 years and 9 months. One month following the final rituximab dose, another attack (TM) manifested, leading to increased lower extremity weakness and loss of bladder control. The CD19+ B-cell count was recorded at 3 cells/µL 1 year and 3 months post-rituximab initiation and dropped to 0 cells/µL just a month before the TM attack.
- Patient 3: Attack (ON and TM) occurred 4 months after initiating rituximab treatment, presenting with continued encephalopathy, worsening lower extremity weakness and symptoms of gait disability, headache, and brain fog. No details of vision changes were provided. CD19+ B-cell count was found to be 3 cells/µL the month of the attack, and 2 cells/µL 3 months prior to the attack.
- Patient 4: Attack occurred 7 months after initiating rituximab treatment, presenting with worsening lower extremity weakness. CD19+ B-cell count was found to be 8 cells/µL 3 months prior to the attack, and 224 cells/µL 7 months prior to the attack (which was prior to initiating therapy).
- Patient 5: Attack occurred 6 months after initiating rituximab treatment, presenting with transverse myelitis and encephalopathy. At time of attack, the patient had a CD19+ B-cell count of 3%. At time of attack, Sagittal STIR, Sagittal T1-post contrast, and Axial T2 MRI imaging were taken, which highlights the transverse myelitis seen approximately 6 months after initiating rituximab treatment (**Figure 1**).
- Patient 6: Attack occurred after 4 years and 3 months on rituximab treatment, presenting as a seizure; confirmed by treating physician and categorized as a breakthrough attack. CD19+ B-cell count was not available at the time.

**S4. Treatment delay/discontinuation (treatment delays recorded: 7 across 4 patients)**

- Patient 1: Note that this is the same patient mentioned in the previous footnote section, “breakthrough disease.” Following 1.5 years of combination therapy involving rituximab (RTX) and MMF, the patient manifested a mild attack characterized by upper extremity weakness. Notably, the last administration of RTX had occurred 8 months preceding this mild attack event, with subsequent treatment involving a rituximab biosimilar commencing 2 months post-attack. The occurrence of the attack raises the possibility of attributing it to treatment delay, stemming from insurance-related issues. Additionally, when the subsequent rituximab dose was scheduled, 6 months post-administration of the rituximab biosimilar (as previously mentioned), the patient encountered yet another treatment delay due to persistent insurance challenges. Consequently, an attack ensued 2 months after the originally scheduled dose. In response, the patient underwent inpatient treatment with plasmapheresis (PLEX), intravenous immunoglobulin (IVIG), and intravenous steroid therapy as rescue interventions. One dose of rituximab (1000 mg) was administered before transitioning to inebilizumab 3 months later. It is important to note that the patient continued MMF throughout the transition and only discontinued it after receiving the second dose of inebilizumab.
- Patient 2: Patient was on a course of rituximab therapy (1000 mg every 6 months) over a period of 5 years. For the next 4 years after, the patient did not receive treatment due to undisclosed reasons. As a result, the patient experienced two recurrent attacks characterized by optic neuritis (ON). These attacks occurred in the final two years (8th and 9th years) of missed treatments; T1 post-contrast coronal and axial MRI imaging were provided, which highlights the patient’s optic nerve enhancement.
- Patient 3: Patient 3 encountered an attack characterized by new active transverse myelitis (TM) lesions after 4 years of initiating rituximab treatment. The attack was attributed to a lapse in treatment due to insurance-related difficulties, with the precise duration of the lapse undisclosed. Treatment with rituximab was resumed 3 months following the attack; however, an additional extended lapse in treatment ensued. Two years after the previous rituximab dose, the patient sought consultation with the current treating neurologist. During this consultation, it was noted that the patient had transitioned to a new insurance provider and, subsequently, discovered that authorization for rituximab had not been granted. The patient declined an alternative treatment option involving MMF. Seven months later, the patient experienced another attack characterized by recurrent enhancing TM lesions spanning T7-T3 and T4-T8 levels, notably occurring after a period of more than 2.5 years without rituximab dosing. MRI scans during this episode encompassed Sagittal T1 Post STIR, Sagittal T1 Post, and Sagittal T2 of the spine. Both Sagittal T2 and Sagittal T1 fat-saturated sequences highlighted cord hyperintensity from T4 to T8, complemented by Sagittal T1 gadolinium sequences that showed cord enhancement within the same segment. The patient received high-dose intravenous corticosteroids during hospitalization, with plasmapheresis (PLEX) being declined. During this hospitalization, the care team observed mood changes in the patient, prompting a psychiatric consultation. Subsequently, the patient received a diagnosis of paranoid delusional disorder, characterized by the belief of being followed by someone. The patient's non-compliance with rituximab treatment may be attributed to concerns regarding her daughter's capacity to tolerate the medication, particularly given her daughter's own diagnosis of Neuromyelitis Optica Spectrum Disorder (NMOSD) and concurrent diagnosis of delusional disorder. Nevertheless, it is noteworthy that the timing of attacks also aligned with challenges related to insurance coverage. Upon hospitalization, the patient received another rituximab dose; however, this marked the final dose, as the subsequent dose scheduled 8 months later was once again denied by insurance. Inebilizumab was subsequently administered 10 months after the final rituximab dose.
- Patient 4: Patient 4 experienced an attack characterized by Area Postrema Syndrome, Ataxia, and Brainstem Syndrome, occurring 4 years following the last administration of rituximab. MRI scans of the brain during this episode include Axial FLAIR, Axial T1 Post Contrast, and Coronal T2; notable MRI findings revealed a novel lesion located in the left cerebellar peduncle. At the time of attack, the CD19+ B-cell count was measured at 21%.

Note: the patient numbers listed in, S3 and S4 do not correlate with each other unless otherwise specified.
